# Time to Start Delivering Iron Chelation Therapy in Newly Diagnosed Severe *β*-Thalassemia

**DOI:** 10.1155/2020/8185016

**Published:** 2020-12-13

**Authors:** Susi Susanah, Ponpon S. Idjradinata, Nur M. Sari, Lulu E. Rakhmilla, Yunia Sribudiani, Jessica O. Trisaputra, Octawyana Moestopo

**Affiliations:** ^1^Department of Child Health, Hematology-Oncology Division, Hasan Sadikin General Hospital/Faculty of Medicine, Universitas Padjadjaran, Bandung 40161, Indonesia; ^2^Department of Public Health, Epidemiology and Biostatistic Division, Faculty of Medicine, Universitas Padjadjaran, Bandung 40161, Indonesia; ^3^Department of Biomedical Sciences, Division of Biochemistry and Molecular Biology, Faculty of Medicine, Universitas Padjadjaran, Bandung 40161, Indonesia; ^4^Faculty of Medicine, Universitas Padjadjaran, Bandung 40161, Indonesia

## Abstract

**Background:**

Iron overload is still a major complication of severe *β*-thalassemia. Indication to start iron chelation therapy is based on serum ferritin (SF) or transferrin saturation (TS) level or the amount of transfusion. The goal of this study is to analyse the pattern of iron status, the amount of transfusion regarding the time to start iron chelator, and serum hepcidin levels in newly diagnosed severe *β*-thalassemia.

**Methods:**

A prospective cohort study was performed at Hasan Sadikin General Hospital on newly diagnosed severe *β*-thalassemia patients. Subjects had not received any blood transfusion with normal liver function test, CRP, and IL-6 levels who consumed normal diet according to age. The SF and TS levels indicate iron status, while hepcidin level indicates iron regulator status. Main indicator to start iron chelation therapy when SF level ≥1.000 ng/mL, TS level ≥70%, or after receiving transfusion at least 10 times. Statistical analysis used Mann–Whitney and Spearman.

**Results:**

Forty-two newly severe *β*-thalassemia, 30 (71.4%), were diagnosed before 1 year old, mean 9.9 ± 6.4 months, range 2–24 months. Range amount of transfusion until SF level reached ≥1,000 ng/mL were 4-12 times, mean 7 ± 2 times. Mean SF and TS level at diagnosis were 365.6 ± 194.9 ng/mL and 67.3 ± 22.5%, while hepcidin level was normal, mean 242.6 ± 58 ng/mL. 36/42 patients have reached SF >1000 ng/mL with amount of transfusion less than 10 times. There was no significant difference of SF, TS, and hepcidin levels when SF >1000 ng/mL in the group with amount of transfusion 7–12 and less than 7 (*p* = 0.454, *p* = 0.084, *p* = 0.765), respectively. A significant positive correlation between SF and amount of transfusion was observed (*p* < 0.001; *r* = 0.781).

**Conclusion:**

Iron overload in severe *β*-thalassemia patients might occur earlier even before they received 10 times transfusion. Hepcidin serum level tends to increase when iron overload just started.

## 1. Introduction

Beta thalassemia is a group of hereditary disorders characterized by reduction or absence of *β*-globin production resulting alpha globin chain accumulation, forming aggregate, which impairs red blood cell (RBC) production that leads to hemolytic anemia and ineffective erythropoiesis (IE) [1]. Ineffective erythropoiesis leads to increased iron absorption in intestine [1, 2]. Beta thalassemia major and severe form of HbE/*β*-thalassemia generally demonstrate as transfusion-dependent thalassemia (TDT) cases causing iron overload which aggravated by chronic transfusion therapy [2].

The main cause of severe *β*-thalassemia complications is iron overload. Excess accumulation of iron in organ (hemosiderosis) leads to oxidative damage as a result of generation of reactive oxygen species (ROS). A remarkable variability of tissue iron distribution has been observed in severe *β*-thalassemia: liver, heart, and endocrine glands are the organs which most severely affected [1, 3]. Humans do not have any special mechanism that effective to eliminate iron overload, so iron balance in the body was regulated by controlling its absorption. When iron reservation is enough, the absorption is decreased on the contrary when iron reservation is low, the absorption will be increasing. Increased iron absorption is expected to be associated with hepcidin as a 25 amino acid peptide that regulates iron homeostasis. Hepcidin is the main regulator of iron homeostasis by controlling iron absorption in the small intestine and iron release from macrophages and liver [4].

Hepcidin is suppressed by hypoxia and iron deficiency and upregulated by inflammation and iron loading [4, 5]. In condition of iron deficiency, low transferrin saturation (TS) suppressed hepcidin expression, but in IE as in severe *β*-thalassemia with high TS levels, hepcidin expression was also found to be suppressed [4]. Decreased hepcidin expression as a result from iron overload in severe *β*-thalassemia is induced by erythropoiesis regulators as a response to IE [6, 7].

Due to the risk of those iron toxicities, it might be countered as earlier as possible. The body iron status can be determined by measuring serum ferritin (SF) and TS levels. Current practice to start iron chelation therapy based on Thalassemia International Federation (TIF) guidelines is when SF levels rise above 1,000 ng/mL or TS level ≥70% or after received of 10–20 times pack red cells (PRC) transfusions [2].

Excess iron in *β*-thalassemia and severe HbE/*β*-thalassemia often develop early, even before the patient received any blood transfusion [4]. Evaluation of iron burden is essential for the determination of clinical outcomes, deciding when to commence chelation, selection of the regime that should be prescribed, the continuous monitoring of chelation efficacy, and the fine-tuning of the regime. Starting relative intensive chelation in younger children may prevent short stature and abnormal pubertal maturation as well as other iron-related morbidities, and significantly improve survival [8–10].

To date, the studies related to the severity of hemosiderosis in severe *β*-thalassemia most reported patients who have had receiving multitransfusions, there was no study reported iron status and hepcidin level in newly diagnosed severe *β*-thalassemia patients. The purpose of this study was to analyse the pattern of the iron status (SF and TS levels) and hepcidin level in newly diagnosed severe *β*-thalassemia patients at first diagnosis and when they reached iron overload, also regarding the time to initiate iron chelator.

## 2. Materials and Methods

A prospective cohort study was carried out on 42 newly diagnosed severe *β*-thalassemia patients at the Department of Child Health Hasan Sadikin General Hospital from October 2011 to March 2013, taken by consecutive sampling.

The main inclusion criteria were (1) confirmed diagnosed of severe *β*-thalassemia, as determined by result of Hemoglobin (Hb) electrophoresis using high-performance liquid chromatography, (2) had never received any blood transfusion, (3) confirmed transfusion-dependency and initiation of regular protocol of PRC transfusion to maintain hemoglobin level (Hb) >9 g/dL, and (4) iron chelation therapy were initiated when SF level >1000 ng/mL. The exclusion criteria were if they had any signs and symptoms of inflammation confirmed by abnormal serum transaminase (AST, ALT), the level of CRP >5 mg/dL, and IL-6 >16.4 pg/mL.

The patients were followed regarding to prove that all subjects clinically need regular transfusion, as TDT cases, and then, they will be monitored for the amount of transfusion as in 10–15 mL/kg of PRC when the Hb level was below 9 g/dL. Blood samples for the measurement of SF and Hb were collected and measured regularly concomitant with blood transfusion. Sampling was done in the morning, under fasting conditions. While hepcidin level and TS were measured only at the first diagnosis was confirmed and as soon as SF level >1,000 ng/mL. TS was measured by dividing serum iron by total iron binding capacity (TIBC). Iron status was assessed by measuring SF and TS using enzyme immunoassays (Centaur XPT, Siemen). Serum hepcidin level was measured by human hepcidin, ELISA Kit (ELISA Reader, Rayto). Dietary intake was obtained by history taking regarding the subject's food-consumption patterns involving exclusive breastfeeding, infant formula, and complementary foods.

The study was approved by the ethical committee of Hasan Sadikin General Hospital, Faculty of Medicine, Universitas Padjadjaran, Bandung, and was conducted according to the principles of the Declaration of Helsinki. Written informed consent for each patient was obtained from the parents prior to any study procedures being carried out. The period of recruitment was 16 months.

Data were entered into Microsoft Excel, cross-checked, and analysed using SPSS version 25.0 (IBM Corp., Armonk, NY). Descriptive results of categorical variables were described as number and percentage, while continuous variables were described as mean ± SD or median (IQR). Spearman's correlation coefficient was used to test the correlation between SF level and the amount of transfusion, while the Mann–Whitney test was used for the comparison between groups of amount of transfusion (7–12 and less than 7). A *p* value < 0.05 was considered as statistically significant in all analyses.

## 3. Results

There were 42 subjects who met the criteria as newly diagnosed severe *β*-thalassemia. Most of them have *β*-thalassemia major (90%), and the rest were severe HbE/*β*-thalassemia (10%). When the diagnosis was confirmed, the age of the subjects was less than 24 months, and 30/42 (71%) were under one year old with equal by gender. Most of the patients admitted to the hospital at first diagnosis with severe anemia. Serum ferritin (SF) and TS level at diagnosis were already high, while hepcidin level was normal. 36/42 (85.7%) subjects already had a high level of SF based on age. At the time of diagnosis, serum iron level has already high while TIBC within normal limit, so most of the subjects 33/42 (78.5%) had high TS level based on age and increased to 39/42 (92.8%) when SF >1000 ng/mL. The high hepcidin level was found in 6/42 (14.2%) when SF >1000 ng/mL. In this study, 36/42 (85.7%) patients have reached SF >1000 ng/mL when the amount of transfusion less than 10 times (Table 1). The majority of our subjects (71.4%) were exclusively breastfed and continued until 2 years old with additional complementary food from 6 months old.

There was no significant difference of SF, TS, and hepcidin levels when SF >1000 ng/mL in the group with amount of transfusion 7–12 and less than 7 (*p* = 0.454, *p* = 0.084, *p* = 0.765), respectively (Table 2).

**Figure 1:**
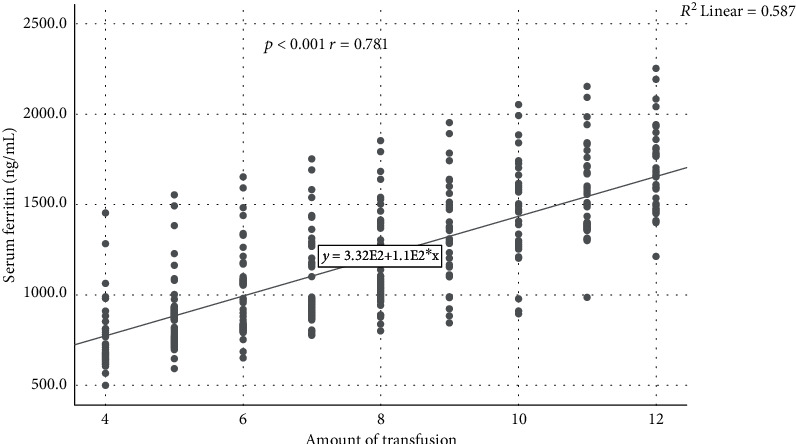
Correlation between serum ferritin level and amount of transfusion (Spearman's correlation). A significant positive correlation was observed in this study (*p* < 0.001, *r* = 0.781).

A significant positive correlation between serum ferritin and amount of transfusion was observed in this study (*p* < 0.001; *r* = 0.781) (Figure 1).

## 4. Discussion

This study showed that most of the subjects were diagnosed under 2 years old; most of them were before 1 year old. This is in line with the characteristic of children with severe *β*-thalassemia who are generally diagnosed before the age of 2 years [2, 11]. Galanello and Origa also reported that severe *β*-thalassemia patients are generally diagnosed between 6 until 24 months [12]. Trehan et al. reported in their study that 52% patients with severe *β*-thalassemia were diagnosed before 1 year of age and one-third presented between 12 and 24 months of age [11]. It indicated that in severe *β*-thalassemia, the genetic imbalance of globin chains causes severe hemolysis and anemia from an early age [2, 12].

The equal gender of the subjects was concordance with thalassemia characteristic as an inherited autosomal recessive disorder following Mendelian rule [2, 13]. Most of the patients were admitted to the hospital with severe anemia similar with Trehan et al. reported that nearly 40% of subjects suffered from severe anemia, with hemoglobin <5.0 g/dL [11]. In *β*-thalassemia major and severe HbE/*β*-thalassemia, excess of unbound *α*-globin chains will aggregate and precipitate adhering to the membrane erythroid precursors. This will cause cellular and membrane damage, apoptosis of erythroid precursors in bone marrow leading to premature death and hence to IE [2, 12]. In *β*-thalassemia, combination of IE of developing erythroid precursor cell and increase hemolysis of mature RBC is the main causes of anemia [14].

Ineffective erythropoiesis in *β*-thalassemia caused increased iron absorption that is regulated by hepcidin mainly from dietary absorption in duodenum, recycled iron from macrophages, and released stored iron from hepatocytes. When iron-deficient hepatocyte produces less or no hepcidin, allowing iron to enter plasma and when iron is abundant, hepcidin will increase to limit further iron absorption and release from stores. The suppressive effect of erythropoiesis on hepcidin in *β*-thalassemia was caused by IE. It is explained that in *β*-thalassemia with iron overload, the hepcidin level was suppressed [15]. In this study, the hepcidin level at diagnosis was still normal (mean 242.6 ± 58.0) but tends to increase. However, only 6 out of 42 (14.3%) patients had high hepcidin level when SF >1000 ng/mL; this might due to the fact that the transfusion inhibits erythropoiesis that will increase hepcidin level [1, 15, 16].

The different response of hepcidin regulation in these two conditions (anemia and hypoxia) may be due to the difference of iron homeostasis regulation on responding to iron level. The existence of high iron circulation level at that time should be due to as a compensation mechanism to hypoxia and increased erythropoiesis. In the newly diagnosed severe *β*-thalassemia patients, the response of hepcidin should be still physiologic until in multitransfused severe *β*-thalassemia patients downregulation of hepcidin occur which causes anomaly. Apparently, hepcidin regulation by hypoxia was overlapped with iron status in the body. Most of iron excess disorder reflected dysregulation in iron status signal or erythroid signal which cause expression of hepcidin inadequate to maintain normal iron homeostasis, and this condition is different between newly diagnosed severe *β*-thalassemia and multitransfused *β*-thalassemia patients [2, 15].

Iron status can be seen from SF and TS level, even though liver iron concentration (LIC) is a gold standard to predict iron loading [17–19]. In this study, most of the subjects had already high SF and TS levels since diagnosis, while the mean of amount of transfusion was less than 10 times. Based on TIF guidelines, TS level ≥70% or SF level >1000 ng/mL for initiating iron chelation therapy, it means the severe *β*-thalassemia patients should be initiated iron chelator earlier even before 10 transfusions. Coates et al. stated that the measurement of SF and TS can be used for iron overload screening. If TS >50% or SF >300 ng/mL on more than one occasion, it should be considered suspicions of iron overload [2, 20].

In this present study, it was found that in the group amount of transfusion less than 7 had higher value of SF, TS, and hepcidin. It could be considered clinically that the subjects had already high SF and TS before and had reached SF >1000 ng/mL with less than 10 times transfusion. A significant positive correlation between SF and amount of transfusion was observed in this study (*p* < 0.001; *r* = 0.781). Intensive transfusion for TDT patients leads to contributing factor for iron loading that proportional to the received blood. A unit 420 mL of PRC contains approximately 200 mg of iron or 0.47 mg/mL of whole blood donor [2, 21].

Normally, sufficient iron intake was needed for children's physiological growth and development. On the contrary, children with TDT should be avoided from high iron intake including iron-fortified food. The contribution of iron from diet is small compared to blood transfusion, only 1–2 mg/day. Most of the subjects in this study receive exclusive breastfeeding and then added complementary foods at the age of 6 months above. It can be predicted that their estimated iron intake and its bioavailability only met their daily physiological requirement and will not contribute to iron overload in the body. This is in line with El Safy et al. study who stated there is no significant difference of iron status in infants with *β*-thalassemia major who received exclusive breastfeeding, exclusive formula feeding, and combination of them. Iron concentration in human milk is lower compared to infant formula, and seemingly, it should not contribute to iron burden in newly diagnosed TDT patients [2, 22]. Moreover, this study showed the newly diagnosed severe thalassemia baby has high iron body level since the beginning, at diagnosis mean SF level was 365.6 ± 194.9 with range (65.2–768.3), while the mean TS level was 67.3 ± 22.5 with range (20.1–98.7) comparing normal SF and TS level in nonthalassemic children (SF 7–140 ng/mL; TS 6–38%), respectively. It seemed that body iron load in newly diagnosed severe thalassemia is mostly due to increased iron absorption related to IE and erythroid expansion mechanism which occurred since the early age [2, 13, 22, 23].

Apparently, iron chelator administration in severe *β*-thalassemia patients should not be only determined by the amount of transfusions. Further study was needed to assign time to initiate iron chelator administration in newly diagnosed severe *β*-thalassemia patients, as nowadays, the medication for them is available. Origa et al. also reported that early chelation therapy in children with TDT should be administered to prevent iron-related complication such as hypogonadism [24]. A randomized controlled trial by Elalfy et al. reported the safety and efficacy of early start of iron chelation therapy in young children newly diagnosed with TDT [25]. A multicentre study from Egypt, Indonesia, and Malaysia and also other studies of Makis et al. (Greece) and Chuansumrit et al. (Thailand) showed safety and efficacy of oral iron chelator in TDT children 1–10 years old [26–28]. Detrimental effects of iron overload can be prevented with good adherences to iron chelation therapy [1, 9, 10].

## 5. Conclusions

Elevated iron status has been existed in newly diagnosed severe *β*-thalassemia, while hepcidin level still normal although the value tends to increase. It is suggested that iron chelator should be administered earlier.

## Figures and Tables

**Table 1:** The characteristic of subjects at diagnosis and at SF level >1000 ng/mL.

Characteristic	At diagnosis (*n* = 42)	At SF level >1000 ng/mL (*n* = 42)
Iron status		
SF (ng/mL); mean ± SD; median (IQR)	365.6 ± 194.9; 319.5 (65.2–768.3)	1,204.4 ± 141.7; 1,175.5 (1,002–1,508)
TS (%); mean ± SD; median (IQR)	67.3 ± 22.5; 70.0 (20.1–98.7)	78.5 ± 17.3; 82.6 (30.1–100.0)
Hepcidin level (ng/mL); mean ± SD; median (IQR)	242.6 ± 58.0; 255.0 (114.5–363.5)	402.7 ± 159.3; 366.6 (176.1–876.0)
Serum iron (*μ*g/dL); mean ± SD; median (IQR)	177.7 ± 77.9; 173 (45–330)	182.4 ± 49.2; 189 (71–261)
TIBC (*μ*g/dL); mean ± SD; median (IQR)	258.7 ± 59.4; 265 (132–367)	233.0 ± 40.6; 230 (153–345)
Amount of transfusion when SF level >1000 ng/mL; mean ± SD; median (IQR)	7.6 ± 2; 8 (4–12)	
Type of thalassemia; *n* (%)		
*β*-Thalassemia mayor	38 (90)	
Severe HbE/*β*-thalassemia	4 (10)	
Age (month); mean ± SD; median (IQR)	9.9 ± 6.4; 7.5(2–24)	
Gender; *n* (%)		
Male	22 (52)	
Female	20 (48)	
Hb (g/dL); mean ± SD; median (IQR)	5.1 ± 1.3; 5.2 (1.6–7.8)	
Dietary status; *n* (%)		
Exclusive breastfeeding		
Yes	42 (100%)	
No	0 (0%)	
Continued breastfeeding until 2 years old		
Yes	30 (71.4%)	
No	12 (38.6%)	
Complementary foods		
Yes	42 (100%)	
No	0 (0%)	

**Table 2:** Comparison between iron status and hepcidin at SF level >1000 ng/ml with group amount of transfusion (*n* = 42).

Variable	Amount of transfusion	Mean ± SD	Median (IQR)	Mean difference	95% CI		*p* value^∗^
					Lower bound	Upper bound	
SF level	1–6	1236.2 ± 155.5	1,214 (1,054–1,491)	46.1	1,142.2	1,330.2	0.454
	7–12	1190.1 ± 135.5	1162.0 (1002–1508)		1,138.6	1,241.6	
TS at SF >1000 ng/mL	1–6	84.1 ± 16.3	84.8 (36.2–100.0)	8.1	74.2	93.9	0.084
	7–12	76.0 ± 17.5	81.8 (30.1–96.1)		69.3	82.6	
Hepcidin at SF >1000 ng/mL	1–6	420.9 ± 193.0	367.9 (176.1–876.0)	26.3	304.2	537.5	0.765
	7–12	394.5 ± 144.7	362.6 (203.1–785.6)		339.5	449.6	

^∗^Mann–Whitney test.

## Data Availability

The data used to support the findings of this study are available from the corresponding author upon request.

## References

[1] Gardenghi S., Gray R. W., Rivella S. (2010). Anemia, ineffective erythropoiesis, and hepcidin: interacting factors in abnormal iron metabolism leading to iron overload in *β*-thalassemia. *Hematology/Oncology Clinics of North America*.

[2] Cappellini M. D., Cohen A., Porter J., Taher A., Viprakasit V. (2014). *Guidelines for the Management of Transfusion Dependent Thalassaemia (TDT)*.

[3] Gardenghi S., Ramos P., Follenzi A. (2010). Hepcidin and Hfe in iron overload in *β*-thalassemia. *Annals of the New York Academy of Sciences*.

[4] El Beshlawy A., Alaraby I., Kader M. S. A., Ahmed D. H., Adelrahman H. E. M. (2012). Study of serum hepcidin in hereditary hemolytic anemias. *Hemoglobin*.

[5] Pasricha S., Frazer D. M., Bowden D. K., Anderson G. J. (2013). Transfusion suppresses erythropoiesis and increases hepcidin in adult patients with *β*-thalassemia major: a longitudinal study. *Blood*.

[6] Nemeth E. (2010). Hepcidin in *β*-thalassemia. *Annals of the New York Academy of Sciences*.

[7] Nemeth E. (2013). Hepcidin and *β*-thalassemia major. *Blood*.

[8] Koren A., Fink D., Admoni O., Tennenbaum-Rakover Y., Levin C. (2010). Non-transferrin bound labile plasma iron and iron overload in Sickle Cell Disease: a comparative study between Sickle Cell Disease and *β* thalassemic patients. *European Journal of Haematology*.

[9] Porter J. B., El-Alfy M., Viprakasit V. (2016). Utility of labile plasma iron and transferrin saturation in addition to serum ferritin as iron overload markers in different underlying anemias before and after deferasirox treatment. *European Journal of Haematology*.

[10] Cappellini M. D., Bejaoui M., Agaoglu L. (2011). Iron chelation with deferasirox in adult and pediatric patients with thalassemia major: efficacy and safety during 5 years’ follow up. *Blood*.

[11] Trehan A., Sharma N., Das R., Bansal D., Marwaha R. K. (2015). Clinicoinvestigational and demographic profile of children with thalassemia major. *Indian Journal of Hematology and Blood Transfusion*.

[12] Galanello R., Origa R. (2010). Beta-thalassemia. *Orphanet Journal of Rare Disease*.

[13] Sankaran V. G., Nathan D. G., Orkin S. H., Orkin S. H., Fisher D. E., Ginsburg D., Look A. T., Lux S. E., Nathan D. G. (2015). Thalassemias. *Hematology and Oncology of Infancy and Childhood*.

[14] Leecharoenkiat K., Lithanatudom P., Sornjai W., Smith D. R. (2016). Iron dysregulation in beta-thalassemia. *Asian Pacific Journal of Tropical Medicine*.

[15] Ganz T., Nemeth E. (2012). Hepcidin and iron homeostasis. *Biochimica et Biophysica Acta*.

[16] Susanah S., Idjradinata P. (2015). Association of *β*-thalassemia type and polymorphisms of c.-582 A>G promotor HAMP gene and iron status in newly diagnosed severe *β*-thalassemia. *Bandung Medical Journal*.

[17] Elsayed M. E., Sharif M. U., Stack A. G. (2016). Transferrin saturation: a body iron biomarker. *Advances in Clinical Chemistry*.

[18] Krittayaphong R., Viprakasit V., Saiviroonporn P., Wangworatrakul W., Wood J. C. (2018). Serum ferritin in the diagnosis of cardiac and liver iron overload in thalassaemia patients real-world practice: a multicentre study. *British Journal of Haematology*.

[19] Sobhani S., Rahmani F., Rahmani M., Askari M., Kompani F. (2019). Serum ferritin levels and irregular use of iron chelators predict liver iron load in patients with major beta thalassemia: a cross-sectional study. *Croatian Medical Journal*.

[20] Coates T. D., Carson S., Wood J. C., Berdoukas V. (2016). Management of iron overload in hemoglobinopathies: what is the appropriate target iron level?. *Annals of the New York Academy of Sciences*.

[21] Danjou F., Cabantchik Z. I., Origa R. (2014). A decisional algorithm to start iron chelation in patients with beta thalassemia. *Haematologica*.

[22] El Safy U. R., Fathy M. M., Hassan T. H. (2017). Effect of breastfeeding versus infant formula on iron status of infants with beta thalassemia major. *International breastfeeding journal*.

[23] Lanzkowsky P., Lipton J. M., Fish J. D. (2016). *Lanzkowsky’s Manual of Pediatric Hematology and Oncology*.

[24] Origa R., Tatti F., Zappu A. (2017). Earlier initiation of transfusional and iron chelation therapies in recently born children with transfusion-dependent thalassemia. *American Journal of Hematology*.

[25] Elalfy M. S., Adly A., Awad H., Tarif Salam M., Berdoukas V., Tricta F. (2018). Safety and efficacy of early start of iron chelation therapy with deferiprone in young children newly diagnosed with transfusion-dependent thalassemia: a randomized controlled trial. *American Journal of Hematology*.

[26] Elalfy M. S., Sari T. T., Lee C. L., Tricta F., El-Beshlawy A. (2010). The safety, tolerability, and efficacy of a liquid formulation of deferiprone in young children with transfusional iron overload. *Journal of Pediatric Hematology/Oncology*.

[27] Makis A., Chaliasos N., Alfantaki S., Karagouni P., Siamopoulou A. (2013). Chelation Therapy with Oral Solution of Deferiprone in Transfusional Iron- Overloaded Children with Hemoglobinopathies. *Anemia*.

[28] Chuansumrit A., Songdej D., Sirachainan N., Wongwerawattanakoon P., Kadegasem P., Sasanakul W. (2016). Safety profile of a liquid formulation of deferiprone in young children with transfusion-induced iron overload: a 1-year experience. *Paediatric and International Child Health*.

